# Weak polarization electric field Ⅲ-N LEDs on polar plane with enhanced efficiency and strong lateral carrier confinement

**DOI:** 10.1038/s41377-026-02359-6

**Published:** 2026-07-01

**Authors:** Jingkai Zhao, Changcai Zuo, Lidong Zhang, Haozhe Gao, Yuliang Liu, Gaoqiang Deng, Xiaohang Li, Yuantao Zhang

**Affiliations:** 1 State Key Laboratory of Integrated Optoelectronics, College of Electronic Science and Engineering, Jilin University, Qianjin Street 2699, Changchun, 130012 China; 2 Advanced Semiconductor Laboratory, King Abdullah University of Science and Technology (KAUST), Thuwal, 23955-6900 Saudi Arabia

**Keywords:** Inorganic LEDs, Optoelectronic devices and components

## Abstract

Weak polarization electric field (PEF) III-nitride LEDs provide a promising pathway toward high-performance micro-LEDs for next-generation displays and wearable devices. To realize this promise, improving the efficiency of weak-PEF LEDs is crucial, as it remains the central requirement for next-generation optoelectronic applications. Here, we demonstrate record-high efficiency weak-PEF LEDs on polar c-plane with InGaN/AlGaN digital alloy (DA) superlattice barriers, enabled by optimized pulse-growth mode of metal-organic chemical vapor deposition (MOCVD). In addition, we reveal strong lateral carrier confinement in these LEDs. We improve the structural quality and optical performance of weak-PEF InGaN/DA multiple quantum wells (MQWs) by optimizing the pulse-growth conditions. Further, we realize weak-PEF DA-based blue LEDs with peak external quantum efficiency up to 15%, which is the highest value in current studies of its kind. Importantly, weak-PEF DA-based LEDs have strong lateral carrier confinement, leading to less efficiency degradation from sidewall effects than conventional GaN-based MQWs LEDs. This work represents a meaningful step toward high-performance weak-PEF III-nitride LEDs, particularly for micro-LED applications where both efficiency and carrier confinement are critical.

## Abstract (web summary)

Wafer-scale weak-PEF LEDs on polar c-plane exhibit the highest EQE of its kind through optimized digital-alloy barrier growth, with great wavelength stability and strong lateral carrier confinement.

## Graphical abstract

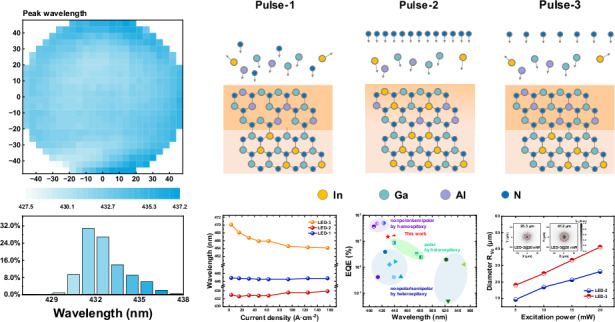

## Introduction

GaN-based light-emitting diodes (LEDs) represent a significant area of interest within the semiconductor optoelectronic industry. Especially, small-sized micro-LEDs can be employed in a multitude of consumer electronic devices, including televisions, computers, mobile phones, and other electronic devices. Additionally, they play a pivotal role in the development of wearable devices, such as augmented reality (AR) and virtual reality (VR) technologies. However, the development of GaN-based LEDs has been constrained by the strong polarization electric field (PEF). Due to the lack of inversion symmetry of GaN along the *c*-axis, there is an inherent polarization effect of *c*-plane (polar plane) GaN. The polarization effect gives rise to the formation of a PEF of the order of MV cm^−1^ in the active region of multiple quantum wells (MQWs), which results in the quantum confined Stark effect (QCSE). Furthermore, the QCSE can further tilt the energy bandgap and cause a spatial separation between the wave-function distributions of holes and electrons. Consequently, a series of adverse effects are thus produced on LEDs, such as the red-shift of the emission wavelength, the decrease in the luminous efficiency, the increase of the carrier lateral diffusion length, and the reduction of modulation bandwidth^1–3^. These effects severely degrade device performance, particularly detrimental to micro-LEDs, which demand exceptional wavelength stability, high efficiency, and strong carrier confinement. Despite these challenges, *c*-plane remains the prevailing orientation for commercial LED production due to its mature epitaxial technology, high crystalline quality, and wafer-scale compatibility. Therefore, it is both technically critical and industrially desirable to mitigate the QCSE on the polar *c*-plane.

Among the strategies developed to mitigate the QCSE, InGaN quantum dots (QDs) have shown considerable promise due to their strong quantum confinement^4,5^. However, their complex and delicate fabrication processes pose substantial challenges for mass production^6^. In comparison, weak-PEF MQWs emerge as a potential alternative, achieving QCSE suppression along with compatibility with standard MOCVD processes. Quaternary InAlGaN, in particular, has been recognized as a pivotal material for implementing weak-PEF MQWs^7–10^. In comparison with ternary nitride materials, InAlGaN offers an additional degree of freedom in adjusting lattice constant, band energy, and polarization. Consequently, it becomes feasible to prepare InAlGaN-based MQWs that are either polarization-matched or possess weak PEF, thereby effectively eliminating or mitigating the bottleneck problem of QCSE^11–14^. However, achieving high-crystalline quality InAlGaN with an atomically smooth surface remains a big challenge. The large discrepancy in bond length and binding energy between InN and AlN leads to different atomic mobility and decomposition temperature. This makes it difficult to optimize the growth conditions of InAlGaN, such as growth temperature and V/III ratio^15,16^. Additionally, the large miscibility gap between InN and AlN leads to phase separation in InAlGaN films, resulting in rough surface morphology and reduced luminescence efficiency of MQWs. Furthermore, the characterization of the InAlGaN composition by standard X-ray diffraction (XRD) methods is complicated due to the lack of a unique solution for the quaternary system. Some researchers have reported improvements in the surface morphology and optical properties by optimizing the growth temperature and composition for InAlGaN films^17–19^. However, these methods cannot resolve the conflicting growth conditions required for both AlGaN and InGaN, leading to significant surface roughness and compositional fluctuation when using traditional methods.

To address the above challenges in InAlGaN material, a viable solution is to substitute quaternary material with digital alloy (DA) material. DA is a form of short-period superlattice composed of binary or ternary alloys with atomic-scale layer thicknesses. It has been shown to be effective in solving crystalline problems in III-nitrides such as InGaN and AlGaN^20–22^. InGaN/AlGaN DA is an ideal substitute to resolve the epitaxial problems of InAlGaN. During the growth process of InGaN/AlGaN DA, In and Al atoms are introduced into the reactor chamber separately. This can reduce the immiscibility and improve crystalline quality. Besides, the average content of DA can be precisely controlled by adjusting the composition and thickness of ternary materials. Recently, we have demonstrated weak-PEF LEDs with InGaN/AlGaN DA as quantum barriers (QBs)^23^. The polarization of DA can be controlled by modifying InGaN and AlGaN compositions. Our simulation results show that the DA QBs offer a higher band offset than InAlGaN QBs, which benefits the carrier confinement in quantum wells (QWs). However, the theoretical advantages of DA QBs have not been fully realized in practice, pointing to challenges in growth. The demonstrated weak-PEF LEDs exhibit lower efficiency compared with conventional GaN barrier LEDs. This is mainly attributed to the inevitable aggregation of In atoms and the relatively low growth temperature during the growth of InGaN/AlGaN DA QBs. These factors could degrade surface morphology and optical properties in MQWs^24^. Thus, it is critical to enable high-performance weak-PEF LEDs by improving the growth approach for InGaN/AlGaN DA and elucidating the underlying growth mechanism.

In this work, we have realized the current highest efficiency weak-PEF LEDs with InGaN/AlGaN DA QBs, enabled by an optimized NH_3_ delivery scheme within the pulse-growth mode of metal-organic chemical vapor deposition (MOCVD). Our results show that this optimized NH_3_ delivery scheme (supplying NH_3_ continuously with a reduced NH_3_ flow rate during the AlGaN growth period) can effectively suppress the incorporation of additional In atoms and prevent the introduction of N vacancies (V_N_). By adopting this epitaxial approach, the luminous intensity of MQWs with DA QBs significantly improves. We propose a growth model to explain the epitaxial growth mechanism of DAs in the pulse-growth mode. Also, we have transferred the pulse-growth method of DAs to industrial mass-production MOCVD and realized industrial-scale preparation of weak PEF LED wafer. The prepared InGaN/DA MQWs LEDs exhibited peak external quantum efficiency (EQE) of up to 15% with weak PEF as low as 0.5 MV cm^−1^ and great wavelength stability. Importantly, weak-PEF LEDs demonstrate strong lateral carrier confinement, featuring carrier lifetimes comparable to InGaN QDs and much shorter lateral diffusion lengths than conventional GaN-based MQWs LEDs. This quantum-well-enabled behavior establishes a viable way to address the challenge of balancing industrial-scale production compatibility and carrier confinement in III-nitride micro-LEDs. This work opens an effective approach for the epitaxy of InGaN/AlGaN DA structure, enabling high-efficiency weak-PEF LEDs and providing a viable pathway toward high-performance micro-LED applications.

## Results

**Fig. 1:**
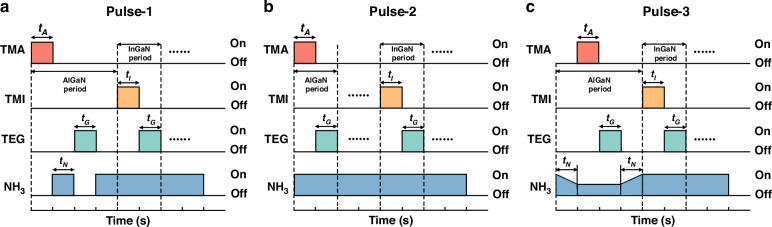
Pulse-growth modes of DAs. **a** Pulse-1, **b** Pulse-2, **c** Pulse-3

In our experiments, we applied three different pulse-growth modes to grow InGaN/AlGaN DAs, which were named as Pulse-1, Pulse-2, and Pulse-3, respectively, as shown in Fig. 1a–c. In the Pulse-1 mode, NH_3_ flow was pulsed during the AlGaN period, while it remained continuous during the InGaN period. In the Pulse-2 mode, NH_3_ continuously passed through both AlGaN and InGaN periods, and its flow rate remained constant. The Pulse-3 mode was like Pulse-2 except that the NH_3_ flow rate in the AlGaN period was reduced. For convenience of description, the values of NH_3_ flow rate in AlGaN period are represented by ${\mathrm{flow}}_{{\mathrm{NH}}_{3}}^{\mathrm{AlGaN}}$. Four samples of DAs, namely DA-1, DA-2, DA-3, and DA-4, were prepared using different pulse-growth conditions described above. While DA-1 to DA-3 directly correspond to Pulse-1 to Pulse-3, DA-4 was also grown using Pulse-3 but with a further reduced NH_3_ flow rate in the AlGaN period. The duration of the TMGa pulse (*t*_G_), TMIn pulse (*t*_I_), and NH_3_ pulse (*t*_N_) was set to 6 s, while the duration of the TMAl pulse (*t*_A_) was adjusted to ensure the same Al content in all samples. The compositions of AlGaN and InGaN in DA are calculated based on XRD measurements. The growth parameters and characterization results can be found in Sections 1 and 2 of the Supplementary Information.

**Fig. 2:**
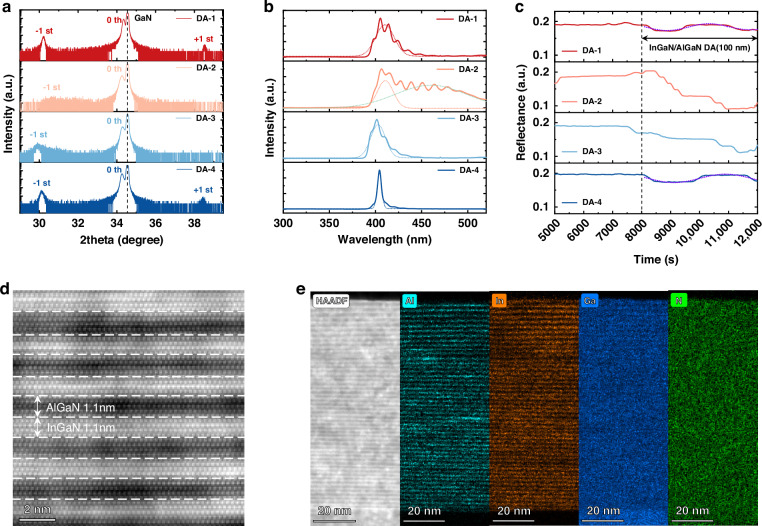
Characterizations of InGaN/AlGaN DAs. **a** XRD 2*θ-ω* scans spectra of the (0002) plane. **b** Room temperature PL spectra at the excitation of 355 nm YAG laser. **c** in-situ monitoring reflectance curves of 405 nm. **d** Cross-sectional STEM image of Sample DA-4. **e** EDS mappings of elemental Al, In, Ga, and N of Sample DA-4

To investigate the structural properties of InGaN/AlGaN DAs, XRD 2*θ-ω* scans spectra of (0002) plane were performed as shown in Fig. 2a. The 0th and −1st satellite peaks related to superlattices as well as the diffraction peaks of GaN templates can be observed. This confirms the periodic structures of DAs. The relationship between the period thickness T and the positions of satellite peaks can be described by the equation^25^:

1 $$T=\frac{\left({n}_{{i}}-{n}_{{j}}\right)\lambda }{2\left(\sin {\theta }_{{i}}-\sin {\theta }_{{j}}\right)}$$

where *λ* is the radiation wavelength of X-ray, *n*_*i*_ and *n*_*j*_ are the orders of satellite peaks, *θ*_*i*_ and *θ*_*j*_ are the positions of satellite peaks. Therefore, the period thicknesses of DA-1, DA-2, DA-3, and DA-4 are determined to be 2.2, 2.5, 2.1, and 2.2 nm, respectively, which are in good agreement with the experimental design values. The average *c*-axis lattice constants obtained from the 0th peaks can be found in Section 2 of the Supplementary Information. Based on the structural parameters, the average In contents of DA-1–DA-4 are determined to be 0.093, 0.126, 0.115, and 0.110, respectively. Among these four samples, DA-1 has the lowest average In content, which could be attributed to the separate introduction of metal-organic sources and NH_3_. For Samples DA-2, DA-3, and DA-4 with continuous ${\mathrm{flow}}_{{\mathrm{NH}}_{3}}^{\mathrm{AlGaN}}$, the average In content decreases as the NH_3_ flow rate decreases. Additionally, the −1st and +1st satellite peaks show obvious variations with the NH_3_ flow rate changing. For DA-1 grown by Pulse-1 mode, both −1st and +1st satellite peaks are clearly distinguished, while those of DA-2 are difficult to discern. As the ${\mathrm{flow}}_{{\mathrm{NH}}_{3}}^{\mathrm{AlGaN}}$ decreases from 0.22 to 0.01 μmol min^−1^, the full width at half maximum (FWHM) of −1st satellite peak reduces from 4777 of DA-2 to 666 arcsec of DA-4. This means a significant improvement in the crystalline quality of the DAs.

To further characterize the compositions and optical properties of InGaN/AlGaN DAs, room-temperature photoluminescence (PL) measurements were performed by a YAG laser (355 nm). As shown in Fig. 2b, all PL spectra exhibit Fabry-Perot resonances due to significant refractive index changes at the sample surfaces and interfaces^26^. Therefore, we use a Gaussian function for spectral fitting to determine the peak wavelengths of the DAs. The emission peaks of DA-1–DA-4 are fitted at 410, 410, 402, and 404 nm, respectively, indicating that the In content in the InGaN layer floats within a narrow range of ±1% for these four samples. It is notable that the spectrum of DA-2 exhibits an additional emission peak at 460 nm with a large FWHM of 77 nm, which gradually disappears as ${\mathrm{flow}}_{{\mathrm{NH}}_{3}}^{\mathrm{AlGaN}}$ decreases from 0.22 μmol for DA-2 to 0.01 μmol for DA-4. The 460 nm peak indicates an accumulation of In atoms in DA-2 with a higher In content than InGaN layers in other DAs. Since the additional peak at 460 nm can be eliminated by NH_3_ modulation during the AlGaN growth period, it can be concluded that the accumulation of In atoms occurs in the AlGaN layer. Besides, a significant reduction in FWHMs of emission peaks is observed as ${\mathrm{flow}}_{{\mathrm{NH}}_{3}}^{\mathrm{AlGaN}}$ decreases. This reduction in FWHM can be attributed to the improvement in structural quality as indicated by XRD results. The results of PL spectra show that reducing the NH_3_ flow rate during the AlGaN period effectively suppresses the luminescence originating from high indium content aggregation, thus enhancing the optical properties of InGaN/AlGaN DAs.

Figure 2c plots the 405 nm in-situ reflectance curves of the DAs growth process. For DA-1, the 405 nm probe light can be reflected by the upper and lower surfaces of DA layers because the band energy of Al_0.16_Ga_0.84_N (3.75 eV) in DAs is higher than the photon energy (3.06 eV) of 405 nm light. The reflectance of DA-1 exhibits Fabry-Perot oscillations related to interference effects. However, the reflectance of DA-2 decreases continuously from 0.20 to 0.09. This significant drop in reflectance (Δ*R* = 0.11) can be attributed to the absorption of 405 nm light, which indicates the reduction in the band energy of DA-2. As the ${\mathrm{flow}}_{{\mathrm{NH}}_{3}}^{\mathrm{AlGaN}}$ decreases from 0.22 for DA-2 to 0.04 μmol min^−1^ for DA-3, Δ*R* decreases from 0.11 to 0.08. For DA-4 with the lower ${\mathrm{flow}}_{{\mathrm{NH}}_{3}}^{\mathrm{AlGaN}}$, Δ*R* completely eliminated, and the reflectance curve shows oscillations similar to DA-1. Based on PL measurement results, it can be concluded that the absorption of 405 nm light in DA-2 and DA-3 results from the higher In content. Figure 2d shows the scanning transmission electron microscopy (STEM) measurement result of DA-4. It can be observed that both InGaN and AlGaN layers are 1.1 nm thick, which is consistent with XRD results. Furthermore, Fig. 2e presents the energy dispersive X-ray spectroscopy (EDS) mapping images of different elements in DA-4. The elemental distributions confirm that the AlGaN and InGaN layers grown by Pulse-3 mode show uniform Al and In incorporation, respectively, and there is no additional diffusion of atoms between the layers.

**Fig. 3:**
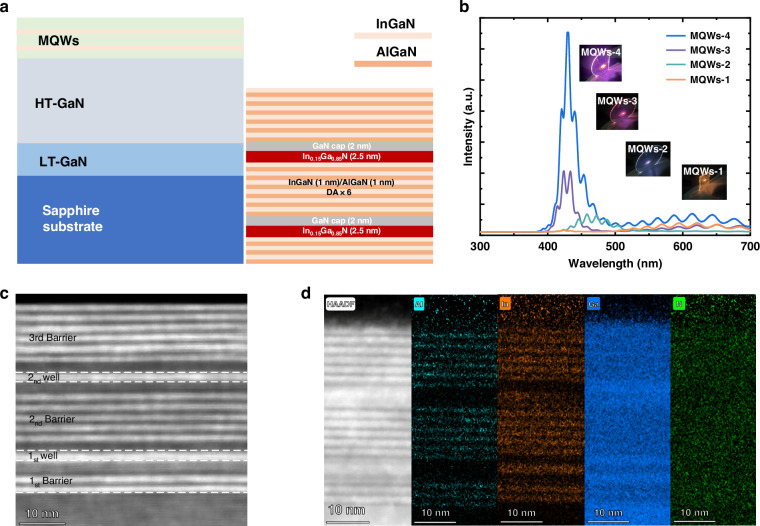
Structure and characterizations of InGaN/DA MQWs. **a** Epitaxy structure of InGaN/DA MQWs. **b** Room temperature PL spectra at the excitation of the He-Cd laser. Insets show luminescence photos of MQWs-1~MQWs-4. **c** Cross-sectional STEM image of Sample MQWs-4. **d** EDS mappings of elemental Al, In, Ga, and N of Sample MQWs-4

Subsequently, four MQWs samples were prepared based on different epitaxial conditions of DAs. They are named as MQWs-1, MQWs-2, MQWs-3, and MQWs-4, using DA-1, DA-2, DA-3 and DA-4 as QBs, respectively. The schematic diagram of the epitaxial structure of MQWs is shown in Fig. 3a. The PL spectra of MQWs at room temperature were characterized using a He-Cd laser (325 nm), as shown in Fig. 3b. It can be observed that the PL intensity of MQWs-1 prepared by Pulse-1 mode is relatively weak, with the only discernible peak of the yellow band. Upon changing the DA growth mode from Pulse-1 to Pulse-2, the PL intensity of MQWs-2 exhibits a significant increase in comparison to that of MQWs-1. The use of Pulse-3 mode with a low ${\mathrm{flow}}_{{\mathrm{NH}}_{3}}^{\mathrm{AlGaN}}$ causes further improvement in the luminous properties of MQWs-3. Further, MQWs-4 grown with the lower ${\mathrm{flow}}_{{\mathrm{NH}}_{3}}^{\mathrm{AlGaN}}$ presents the strongest PL intensity. The poor optical properties of MQWs-1 might be due to the unbalanced stoichiometry in the AlGaN growth period of Pulse-1 mode. The imbalance leads to the formation of V_N_ and C_N_ defects, which increases the non-radiative recombination in MQWs-1^27^. Compared with MQWs-1, the stronger luminous intensity of MQWs-2 indicates that the continuous NH_3_ flow rate in Pulse-2 mode effectively suppresses the formation of N-related defects. Furthermore, the enhanced PL intensity of MQWs-3 and MQWs-4 grown by Pulse-3 can be attributed to the improved structural quality of DA QBs. The emission peaks for Samples MQWs-1~MQWs-4 are located at 427, 455, 430, and 431 nm, respectively. It is notable that the peak wavelength of MQWs-2 (455 nm) shows a significant red-shift. The PL spectrum of DA-2 shown in Fig. 2b exhibits a broad shoulder emission peak at about 460 nm, which corresponds to the emission peak of MQWs-2 at 455 nm. Therefore, the red-shifted emission peak of MQWs-2 might be due to the diffusion of In atoms in the AlGaN layer. Additionally, Fig. 3c, d shows the STEM measurement and EDS mapping of MQWs-4, respectively. The structure of MQWs is in good accordance with the design in Fig. 3a. The DA QBs also show uniform atom distribution and a clear interface in MQWs.

**Fig. 4:**
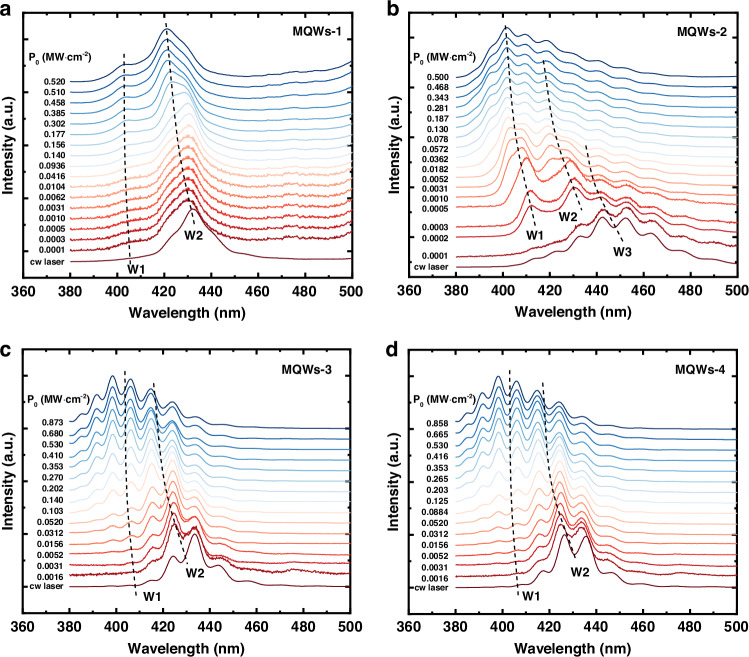
Power-dependent photoluminescence of InGaN/DA MQWs. PDPL spectra of **a** MQWs-1, **b** MQWs-2, **c** MQWs-3, and **d** MQWs-4

To further investigate the luminous properties of MQWs, we performed power-dependent PL (PDPL) measurements at 10 K for MQWs-1, MQWs-2, MQWs-3, and MQWs-4, as shown in Fig. 4a–d. Two emission peaks, named W1 (~405 nm) and W2 (~430 nm), are observed for MQWs-1, MQWs-3, and MQWs-4. Both peaks show a blue-shift with increasing excitation power density, which can be attributed to the carrier screening effect and band-tail filling effect. Based on the PL results in Figs. 2b and 3b, W1 corresponds to the luminescence of DA QBs, while W2 represents the luminescence of InGaN QWs. For MQWs-2, it can be observed that there exists one dominant luminescence peak of W3 at around 450 nm as the excitation power is lower than 2 × 10^−6 ^MW cm^−2^. With the excitation power increasing to 3 × 10^−4^ MW cm^−2^, the peaks of W1 (~405 nm) and W2 (~430 nm) appear, while W3 gradually disappears with increasing power density. The variation of W3 with different excitation power indicates that it is associated with the localized state of In accumulation area. Under lower excitation power density, most of the excited carriers get trapped in the localized states, and thus the lower energy peak (W3) dominates. When the excitation power increases, more photogenerated carriers are injected into QWs. Therefore, the higher energy peaks (W1 and W2) dominate gradually with the increase of the excitation power. Besides, the peak positions of W1 and W2 in MQWs-2 are close to those of other MQWs. This indicates that the In contents in InGaN layers of QWs and QBs are identical in all MQWs samples, and the additional In atoms accumulation occurs in the AlGaN layer. Since the key difference among these MQWs samples is the NH_3_-related growth condition during the AlGaN period in DA, this additional accumulation of In atoms is strongly correlated with the NH_3_ condition. The TEM images of DA-2 grown under the highest NH_3_ flow rate (provided in Section 3 of the Supplementary Information) and DA-4 grown under the lowest NH_3_ flow rate (shown in Fig. 2d) further correlate the NH_3_ condition with the interface quality. It can be found that the interfaces in DA-2 appear considerably ambiguous, whereas DA-4 exhibits clear and well-defined interfaces. Taken together, these comparisons suggest that the NH_3_ flow rate influences the interface quality in association with the In incorporation in the AlGaN layer, which supports our interpretation of the degraded optical performance of MQWs-2.

**Fig. 5:**
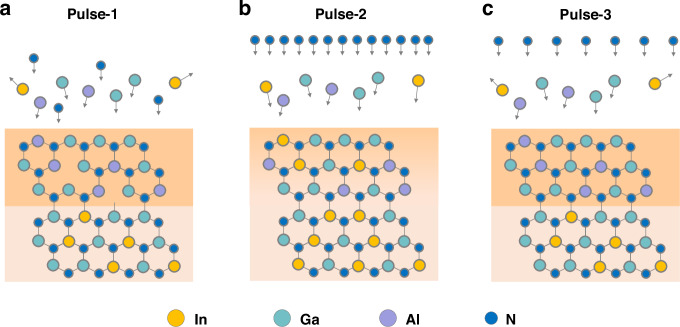
Schematic diagram of the growth model of InGaN/AlGaN DAs. **a** Pulse-1 mode, **b** Pulse-2 mode, and **c** Pulse-3 mode

Based on the experimental results, we propose the growth models for DAs with different pulse modes, as illustrated in Fig. 5. In Pulse-1 mode, NH_3_ and metal-organic source are separately injected into the chamber during AlGaN period, as shown in Fig. 1a. Under the condition of low N atom density, the In atoms are more likely to be desorbed rather than incorporated into the lattice due to the relatively low bond energy of In-N^9^. The suppression of In incorporation actually contributes to the formation of sharp interfaces of DA-1. However, due to the pulse injection of NH_3_, there is a shortage of N atoms during the growth process, resulting in the generation of N-related defects such as V_N_ and C_N_ defects. These defects can serve as non-radiative recombination centers to reduce the optical performance of MQWs-1^26^. As the pulse-growth mode changes from Pulse-1 to Pulse-2, the molar fraction of supplied NH_3_ increases significantly. This provides sufficient N atoms during AlGaN growth, which helps to reduce the density of N-related defects, as shown in Fig. 5b. However, with the diffusion of In atoms, the interfaces of DAs become obscure, resulting in the low PL intensity of MQWs-2. For Pulse-3 mode, the reduction of ${\mathrm{flow}}_{{\mathrm{NH}}_{3}}^{\mathrm{AlGaN}}$ inhibits the bonding of the In atom in the AlGaN period. Meanwhile, the continuous injection of NH_3_ provides a stable growth atmosphere, thereby suppressing the generation of N-related defects and improving the optical properties and structural quality of DAs.

**Fig. 6:**
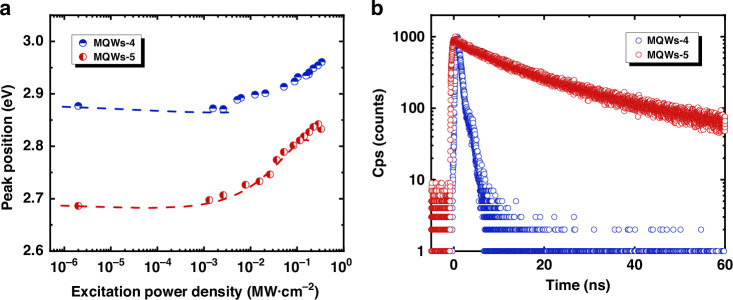
Characterization of MQWs-4 and MQWs-5. **a** PL peak position dependences on excitation power density, the dashed lines demonstrate the theoretical fit of the peak position on excitation power density. **b** Low-temperature TRPL spectra at the PL peak emission wavelength

To demonstrate the influence of Al_0.16_Ga_0.84_N/In_0.19_Ga_0.81_N DAs on the PEF of MQWs, we compared MQWs-4 with conventional InGaN/GaN MQWs-5. Both MQWs have the same QB and QW thicknesses. PDPL measurements were performed for these two samples. Figure 6a shows the position of the emission peak as a function of excitation power density. At a low power density of ~10^-6 ^MW cm^−2^, the peak position of MQWs-4 exhibits a 0.2 eV blue-shift compared with MQWs-5. In addition, the blue-shift of MQWs-4 is smaller as the power density increases from ~10^−6^ to 10^−1^ MW cm^−2^. We use a transition energy model to fit the power-dependent PL data, and the detailed calculations can be found in our previous report^22^. The PEFs of MQWs-4 and MQWs-5 are calculated to be 0.5 and 2.0 MV cm^−1^, respectively. That indicates the Al_0.16_Ga_0.84_N/In_0.19_Ga_0.81_N DA QBs can effectively reduce the PEF in MQWs. Figure 6b shows the results of time-resolved PL (TRPL) at 10 K. To investigate the excitonic dynamics, we use a standard two-exponential component model to fit the experimental data^28^. The fast decay times *τ*_*fast*_ are estimated to be 0.69 and 12.6 ns for MQWs-4 and MQWs-5, respectively, indicating more overlap of electron and hole wavefunctions in MQWs-4^29^. These results prove that the prepared DA QBs effectively decrease the PEF in MQWs compared with conventional GaN QBs. It leads to the improvement of electron and hole wavefunctions’ overlap and the increase of carrier recombination probability.

**Fig. 7:**
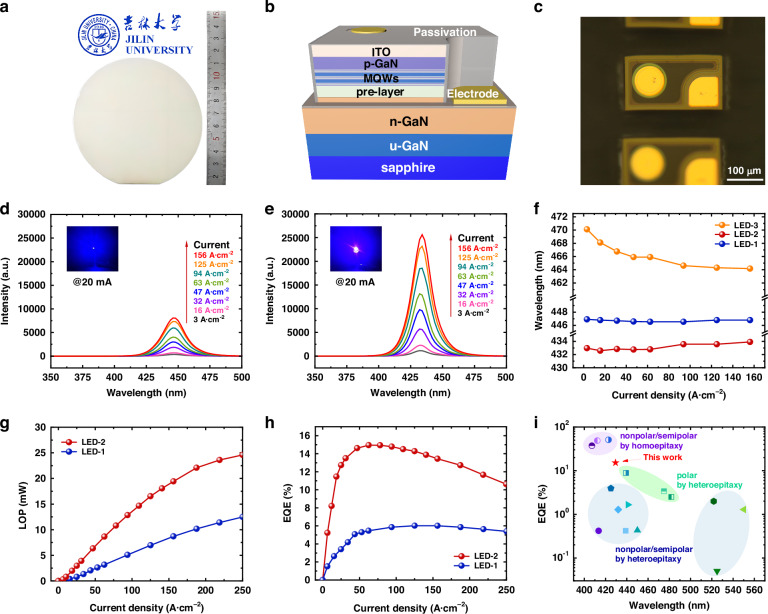
Characterization of LEDs with InGaN/DA MQWs (LED-1, LED-2) and InGaN/GaN MQWs (LED-3). **a** Photograph of the 4-inch DA-based LED wafer. **b** Schematic diagram of the DA-based LED device. **c** Top-view optical microscope image of the DA-based LED device. **d** EL spectra of LED-1. **e** EL spectra of LED-2. The insets show the packaged sample luminescence photo at a current of 20 mA. **f** Peak wavelength changes with current density. **g** Light output power dependences on inject current density. **h** EQE dependences on inject current density. **i** Comparison of the peak EQE of LED-2 and weak PEF LEDs in other reports

Further, we have realized 4-inch industrial-scale preparation of weak-PEF InGaN/DA MQWs LEDs using industrial mass-production MOCVD (Veeco K465I). To verify the effects of optimized DA growth conditions, we prepared two types of weak-PEF LEDs, namely LED-1 and LED-2. The DA QBs of the LED-1 epitaxial structure adopt Pulse-2 mode with a ${{flow}}_{{{NH}}_{3}}^{{AlGaN}}$ of 20 slm, and the DA QBs of the LED-2 epitaxial structure adopt Pulse-3 mode with a reduced ${{flow}}_{{{NH}}_{3}}^{{AlGaN}}$ of 5 slm. Also, an LED-3 with conventional InGaN/GaN MQWs was prepared for comparison with weak-PEF LEDs. The essential structural information distinguishing these three LED samples is summarized in Section 2 of the Supplementary Information. Figure 7a–c shows the 4-inch LED wafer, the schematic diagram of the LED device, and the top-view optical microscope image of the LED device with a size of 147 × 264 μm², respectively. The relevant wavelength mappings of three LEDs are provided in Section 4 of the Supplementary Information. The electroluminescence (EL) spectra at the different injection current densities of LED-1 and LED-2 are shown in Fig. 7d, e, respectively. It can be observed that the peak intensity of LED-2 is significantly higher than that of LED-1, which could be attributed to the suppression of In atoms diffusion. The emission peak wavelengths of LEDs at the different injection current densities are shown in Fig. 7f. At the same current density, the peak wavelength of LED-3 is longer than those of LED-1 and LED-2. This is consistent with the PDPL results. In addition, three LEDs show different wavelength blue-shift with the increase of current density. The maximum blue-shift of LED-3 is 5.9 nm, while those of LED-1 and LED-2 are 0.4 and 0.3 nm, respectively. The smaller blueshifts of LED-1 and LED-2 indicate their weak PEF. Figure 7g, h shows the light output power (LOP) and EQE of the LED-1 and LED-2. For reference, the EL spectra, LOP, and EQE of LED-3 are provided in Section 5 of the Supplementary Information. The LOP of LED-2 is 25.0 mW at 250 A cm^−2^, an increase of 97% compared to that of LED-1. Moreover, LED-2 shows a peak EQE of 15%, and its EQE is still higher than 10% at 250 A cm^−2^. The EL results prove that the optimized DA QBs growth conditions can effectively improve the EQE of weak-PEF LEDs. The peak EQE of LED-2 is compared with other reported values of weak-PEF LEDs, as shown in Fig. 7i^29–43^. It can be observed that LED-2 exhibits the highest EQE (15%) among previously reported heteroepitaxial weak-PEF LEDs grown on polar plane (maximum 8.8%) and nonpolar/semipolar plane (maximum 3.9%). It is only lower than weak-PEF LEDs grown on nonpolar/semipolar planes by homoepitaxy. In this context, although homoepitaxial nonpolar/semipolar weak-PEF LEDs can achieve higher EQE, their reliance on expensive substrates and limited process compatibility makes them less favorable for manufacturing. Taken together, our approach provides a manufacturable weak-PEF route that retains polar c-plane compatibility within heteroepitaxial platforms.

**Fig. 8:**
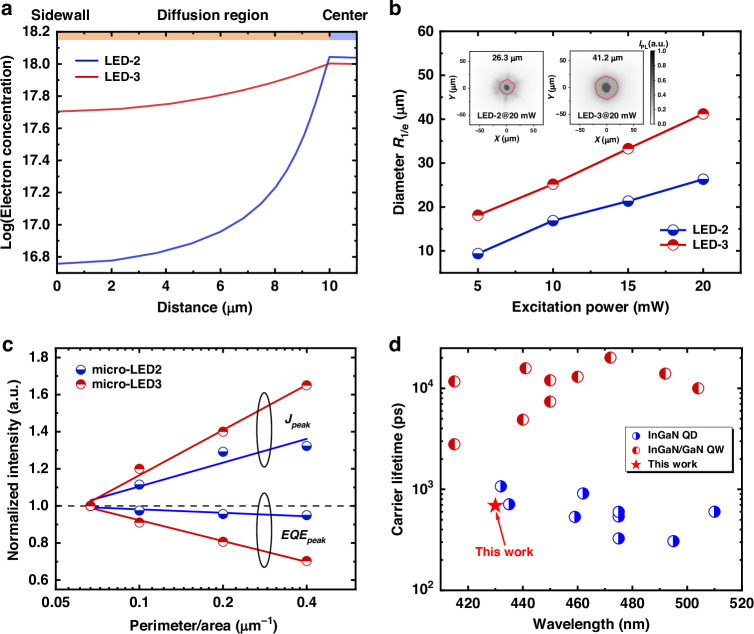
Characterization of LED-2 and LED-3. **a** Simulated lateral electron concentration distribution in QWs of an LED. **b** Micro-PL spot diameter changes with excitation power. Insets show the micro-PL spot (red line denotes the 1/e intensity contour) at an excitation power of 20 mW. **c** Normalized EQE_peak_ and *J*_peak_ dependences on the ratio of perimeter to area for micro-LED-2 and micro-LED-3. Both EQE_peak_ and *J*_peak_ are normalized to the value of the largest size. **d** Comparison of the carrier lifetime measured from MQWs of the weak-PEF LED with conventional InGaN/GaN QWs and InGaN QDs in other reports

In addition to achieving relatively high EQE, the weak-PEF LED with InGaN/DA MQWs also exhibits a strong carrier confinement capability. The lateral diffusion length *L* can be obtained by the equation^44^:

2 $$L=\sqrt{D\tau }$$

where *D* is the carrier diffusion coefficient, *τ* is the carrier lifetime. Therefore, the weak-PEF LED is expected to exhibit a shorter carrier lateral diffusion length than the conventional InGaN/GaN LED, based on the TRPL results shown in Fig. 6b. To clarify the carrier confinement capability of the weak-PEF LED, we use the APSYS software to simulate the lateral distribution of electron concentration in the quantum wells of LED-2 and LED-3, as shown in Fig. 8a. In the simulation, the PEF and carrier lifetime used are based on the measurement results. As electrons diffuse from the LED center (at 10 μm in Fig. 8a) to the sidewall (at 0 μm in Fig. 8a), the electron concentrations of the two LEDs are reduced to different extents. The electron concentrations are reduced by 94% for LED-2 and 50% for LED-3, respectively. Similarly, the corresponding lateral hole concentration distribution is provided in Section 6 of the Supplementary Information, where the hole concentration decreases from the LED center to the sidewall by 78% in LED-2 and 40% in LED-3. It indicates a higher carrier recombination velocity, i.e., a shorter lateral diffusion length, for LED-2. To visually characterize the lateral diffusion of carriers in LEDs, we performed micro-PL measurements on LED-2 and LED-3. Figure 8b shows the micro-PL spot diameter (1/e normalized PL intensity) as a function of the excitation power, while the inset illustrates the micro-PL images of both LEDs at an excitation power of 20 mW. Complete sets of micro-PL images for both LEDs are provided in Section 7 of the Supplementary Information. It can be observed that the spot diameter of both LEDs increased with increasing excitation power. This trend could be attributed to the increase in the carrier diffusion coefficient^45^. It is noteworthy that the spot diameter of LED-2 is considerably smaller than that of LED-3 at all excitation powers. This finding is consistent with the anticipated reduction in carrier diffusion length, attributed to the decreased carrier lifetime. Then, we investigated the impact of carrier lateral diffusion length on the performance of LEDs. To this end, micro-LEDs (micro-LED-2 and micro-LED-3) with different sizes of 10 × 10, 20 × 20, 40 × 40, and 60 × 60 μm² are fabricated based on the structures of LED-2 and LED-3, respectively. Figure 8c shows the size-dependent changes in the peak EQE (EQE_peak_) and the current density at the peak EQE (*J*_peak_) for micro-LED-2 and micro-LED-3. The measurement current density range used for micro-LEDs is identical to that used in Fig. 7h (3–250 A cm^−2^). The Quantitative EQE_peak_ and *J*_peak_ values for both micro-LEDs are provided in Section 8 of the Supplementary Information. The EQE_peak_ and *J*_peak_ of LEDs can be analyzed using the ABC model^46^:

3 $${{EQE}}_{{peak}}=\frac{B}{B+2\sqrt{AC}}\times {\eta }_{e}$$

4 $${J}_{peak}{=}qwA\left(\frac{B}{C}{+}{2}\sqrt{\frac{A}{C}}\right)$$

where *A* is the Shockley–Read–Hall non-radiative recombination rate, *B* is the radiative recombination rate, *C* is the Auger recombination rate, *η*_e_ is the light extraction efficiency, *q* is the elementary charge, and *w* is the total thickness of QWs. Given the independence of *B* and *C* from the LED size^47^, it can be concluded that EQE_peak_ and *J*_peak_ are functions of *A* as the LED size varies. The relationship between *A* and LED size can be described by the equation:

5 $$A={A}_{0}+{V}_{s}\frac{l}{S}$$

where *A*_0_ is the non-radiative recombination rate in the bulk material, *V*_s_ is the surface recombination velocity, *l* is the device perimeter, and *S* is the device area. As demonstrated in the above equation, *A* increases as the ratio of *l* to *S* increases (i.e., device size decreases). This leads to a decrease in EQE_peak_ and an increase in *J*_peak_, which is consistent with the results shown in Fig. 8c. Moreover, Fig. 8c reveals that the size dependence of EQE_peak_ and *J*_peak_ of micro-LED-2 is considerably less than that of micro-LED-3. It should be noted that the *η*_e_ term in Eq. 3 is reported to increase as the micro-LED size decreases^48,49^, which offers an opposite contribution to EQE_peak_ compared to the *A* term. Although the decrease in EQE_peak_ with device size decreasing indicates that it is dominated by the increasing of *A*, it is difficult to quantify sidewall sensitivity through EQE_peak_ with size-dependent *η*_e_. Since *η*_e_ is not included in Eq. 4, the sidewall sensitivity is quantified using the linear-fit slope of the *J*_peak_-*l*/*S* dependence in Fig. 8c. A smaller slope indicates that the micro-LED is less sensitive to the sidewall effect. The extracted slope ratio between micro-LED-2 and micro-LED-3 is approximately 0.5, which is close to the micro-PL spot-diameter ratio shown in Fig. 8b. This result suggests that the weak-PEF LED exhibits an approximately 50% reduction in sidewall sensitivity compared with the conventional LED. Such a characteristic is crucial for micro-LEDs, where strong carrier confinement contributes to mitigate efficiency losses induced by sidewall effects. Additionally, the carrier lifetime measured from MQWs of the weak-PEF LED is compared with reported values of conventional InGaN/GaN QWs and InGaN QDs, as shown in Fig. 8d^47,50–63^. Also, it can be observed that weak-PEF InGaN/DA MQWs exhibit a lower carrier lifetime than conventional InGaN/GaN QWs. Notably, the carrier lifetime is comparable to that of InGaN QDs. This comparison suggests that weak-PEF InGaN/DA MQWs provide an MQW-based route to achieve QD-comparable lifetimes, which is promising for micro-LEDs and LDs where reduced lateral carrier diffusion is desired.

## Discussion

In summary, we demonstrate weak-PEF DA-based LEDs with the current highest EQE and strong lateral carrier confinement, representing a clear performance breakthrough over previous DA-based weak-PEF designs. This enhanced performance is enabled by optimized pulse-mode MOCVD growth of InGaN/AlGaN DA. It is found that pulsed NH_3_ flow rate during AlGaN growth leads to poor luminescence characteristics of InGaN/DA MQWs. Conversely, continuous high NH_3_ flow rate leads to the incorporation of additional In atoms, resulting in inferior structural quality of DA. These issues can be effectively addressed by using an NH_3_ flow rate modulated pulse-growth mode, which suppresses the generation of N-related defects and In clusters in AlGaN. Based on the structure and optical properties of DAs, we propose a growth model to explain the effects of pulse-growth mode on epitaxial growth mechanisms. The prepared InGaN/DA MQWs LEDs exhibited peak EQE of up to 15% with weak PEF as low as 0.5 MV cm^−1^ and great wavelength stability, indicating the superiority of high-quality InGaN/AlGaN DAs for achieving high-performance weak-PEF LEDs. Meanwhile, InGaN/DA MQWs LED exhibits a strong lateral carrier confinement capability, leading to less efficiency degradation from sidewall effects than conventional GaN-based MQWs LEDs and presenting great potential for addressing the challenge of balancing industrial-scale production compatibility and carrier confinement in micro-LEDs. This work proposes a reliable epitaxy approach for preparing high-quality InGaN/AlGaN DAs, and provides an experimental basis for realizing high-performance weak-PEF III-N LED based on the polar plane.

## Materials and methods

### Epitaxial growth of InGaN/AlGaN DAs, MQWs, and LEDs

InGaN/AlGaN DAs were grown on sapphire substrates using the AIXTRON 3×2″ FT MOCVD system. The used precursors were trimethylgallium (TMGa), trimethylaluminum (TMAl), trimethylindium (TMIn), and ammonia (NH_3_) for Ga, Al, In, and N, respectively. H_2_ and N_2_ were used as carrier gases. Prior to deposition, sapphire substrates were cleaned thermally in H_2_ atmosphere at 1100 °C for 5 min. The growth process began with a low-temperature GaN buffer layer grown at 540 °C. Then, the growth temperature was increased to 1050 °C to grow a 2 μm thick undoped GaN template layer. Subsequently, InGaN/AlGaN DAs were grown on the GaN template. The DAs were composed of 70 pairs of ~1 nm thick InGaN QW layers and ~1 nm thick AlGaN QB layers, both of which were grown at 720 °C.

The growth of InGaN/DA MQWs was also prepared on the aforementioned GaN template. The MQWs comprised two pairs of 2.5 nm thick In_0.15_Ga_0.85_N QW layers and 12 nm thick InGaN/AlGaN DA QBs. The DA QBs included six pairs of InGaN/AlGaN superlattices, each consisting of ~1 nm InGaN layer and ~1 nm AlGaN layer. The QW and DA QBs were grown at 750 and 720 °C, respectively.

InGaN/DA MQWs LEDs were prepared on patterned sapphire substrate by an industrial Veeco K465I 14 × 4″ MOCVD system. After the growth of a 2 μm GaN template, a 1 μm n-GaN layer was grown on it at 1050 °C. Then, the growth temperature dropped to 880 °C for the growth of n-In_0.03_Ga_0.97_N/GaN pre-layer. The pre-layer consisted of 3 pairs of 5 nm thick n-In_0.03_Ga_0.97_N well and 40 nm n-GaN barrier. After that, InGaN/DA MQWs were grown on the n-pre-layer; the structure was the same as that of InGaN/DA MQWs described above. A 40 nm p-GaN layer was grown on the last barrier of InGaN/DA MQWs at 720 °C to protect the MQWs. Subsequently, the growth temperature ramped up to 940 °C for the growth of a 20 nm p-Al_0.3_Ga_0.7_N electron blocking layer, a 150 nm p-GaN layer, and a 20 nm p+-GaN ohmic contact layer, respectively. At last, the epilayer was annealed at a temperature of 850 °C for 20 min.

### Fabrication of LEDs

At first, an indium tin oxide (ITO) layer was spin-coated on the surface of the epitaxial wafer as a current spreading layer. Next, UV lithography and inductive coupled plasma (ICP) etching techniques were applied to define the LEDs with different sizes. Then, Cr/Al/Ti/Au (50/200/50/200 nm) were deposited as n- and p-pad after growing 600 nm silicon oxide as the sidewall passivation layer and current isolation layer.

### Characterization

The structure parameters of InGaN/AlGaN DAs and InGaN/DA MQWs were measured by a Rigaku Ultima IV XRD with Cu-Kα radiation (0.154056 nm) and a scanning transmission electron microscope (Jeol-F200). The power-dependent PL spectra of DAs and MQWs were measured by a PL system that includes an IK3501-G He-Cd laser (325 nm, 1.8 W cm^−2^) and a Surelite II-10 YAG laser (355 nm, variable-power laser) as excitation sources, a Jobin Yvon iHR550 spectrometer, a Syncerity charge-coupled device, and a closed-circle helium cryostat. The TRPL measurements were performed using a semiconductor pulsed laser with an excitation wavelength of 405 nm. The signal was dispersed by an iHR 320 spectrometer and detected by a PMA Hybrid 40 single photon counting module. The electroluminescence (EL) characteristics were evaluated using a UATEK EL testing system. The micro-PL spots of LEDs were measured by a micro-PL system, which includes a Coherent OBIS laser (375 nm) as an excitation source, a microscope objective lens (50×, 0.42 numerical aperture), and a CMOS camera sensor.

## Supplementary information

Supplementary Information for Weak Polarization Electric Field Ⅲ-N LEDs on Polar Plane with Enhanced Efficiency and Strong Lateral Carrier Confinement

## Data Availability

The data that support the findings of this study are available from the corresponding authors upon request.
