# Supplementary material for: Weak polarization electric field Ⅲ-N LEDs on polar plane with enhanced efficiency and strong lateral carrier confinement
Source: Light Sci Appl. 2026 Jul 1;15:300. doi: 10.1038/s41377-026-02359-6 (PMC13324002; doi:10.1038/s41377-026-02359-6)
Supplement: Supplementary file 1 — Supplementary Information for Weak Polarization Electric Field Ⅲ-N LEDs on Polar Plane with Enhanced Efficiency and Strong Lateral Carrier Confinement [file 41377_2026_2359_MOESM1_ESM.docx]

**Supplementary Information for**

**Weak Polarization Electric Field Ⅲ-N LEDs on Polar Plane with Enhanced Efficiency and Strong Lateral Carrier Confinement**

Jingkai Zhao^1^, Changcai Zuo^1^, Lidong Zhang^1^, Haozhe Gao^1^, Yuliang Liu^1^, Gaoqiang Deng^1*^, Xiaohang Li^2*^, and Yuantao Zhang^1*^

^1^State Key Laboratory of Integrated Optoelectronics, College of Electronic Science and Engineering, Jilin University, Qianjin Street 2699, Changchun 130012, China

^2^King Abdullah University of Science and Technology (KAUST), Advanced Semiconductor Laboratory, Thuwal 23955-6900, Saudi Arabia

These authors contributed equally: Jingkai Zhao, Changcai Zuo

*Correspondence: Yuantao Zhang (zhangyt@jlu.edu.cn) or

Xiaohang Li (xiaohang.li@kaust.edu.sa) or Gaoqiang Deng (denggq@jlu.edu.cn)

1. **Characterization of AlGaN film**

The compositions of AlGaN in DAs were calculated from the XRD measurements as shown in Fig. S1. Since all of the AlGaN films grown on GaN template are under strain condition, the composition calculation results of XRD are adjusted by the method described in Ref. 1. The AlGaN related diffraction peakd are located in 34.94°, 35.02°, 34.99°, and 34.92° for sample AlGaN-1, AlGaN-2, AlGaN-3, and AlGaN-4, respectively.

**Fig. S1.** XRD 2θ-ω scans spectra of (0002) plane for AlGaN-1, AlGaN-2, AlGaN-3, and AlGaN-4.

1. **Growth and structure parameters of DAs and LEDs**

**Table S1.** Growth parameters of InGaN/AlGaN DAs samples

| Sample | t_N_ (s) | t_A_ (s) | Pulse-growth mode | AlGaN | | | InGaN | | |
| --- | --- | --- | --- | --- | --- | --- | --- | --- | --- |
|  |  |  |  | NH_3_ flow rate (mol·min^-1^) | Al content | Thickness  (nm) | NH_3_ flow rate (mol·min^-1^) | In content | Thickness  (nm) |
| DA-1 | 6 | 3 | Pulse-1 | 0.22 | 0.16 | 1 | 0.22 | 0.19 | 1 |
| DA-2 | — | 6 | Pulse-2 | 0.22 | 0.18 | 1 | 0.22 | 0.19 | 1 |
| DA-3 | 6 | 5 | Pulse-3 | 0.04 | 0.18 | 1 | 0.22 | 0.19 | 1 |
| DA-4 | 6 | 3 | Pulse-3 | 0.01 | 0.16 | 1 | 0.22 | 0.19 | 1 |

The epitaxial growth parameters of DAs were systematically summarized in Table S1. It should be noted that the composition and thickness presented in Table S1 were designed values. The structural characterization data of DAs obtained through XRD measurements were compiled in Table S2. The c-axis lattice constants were quantitatively determined by analyzing the zero-order satellite peaks in (0002) 2θ-ω scans, while the period thicknesses of DAs were derived from the angular positions of adjacent satellite peaks using the modified Bragg equation. Furthermore, the essential structural information of the LED samples is summarized in Table S3. Table S3 summarizes the key structural parameters distinguishing the three LED designs, including their respective barrier structures and DA growth modes, while the quantum well conditions remain identical.

**Table S2.** Structure parameters of InGaN/AlGaN DAs samples

| Sample | c-axis constant (Å) | t (nm) | Calculated In content | FWHM of -1th satellite peak (arcsec) |
| --- | --- | --- | --- | --- |
| DA-1 | 5.216 | 2.2 | 0.093 | 392 |
| DA-2 | 5.229 | 2.5 | 0.126 | 4777 |
| DA-3 | 5.225 | 2.1 | 0.115 | 1256 |
| DA-4 | 5.226 | 2.2 | 0.110 | 666 |

**Table S3.** Growth and structure parameters of LED samples

| Sample | Barrier | Pulse-growth mode of DAs | NH_3_ flow rate in AlGaN period of DAs (slm) | In Content of Well |
| --- | --- | --- | --- | --- |
| LED-1 | InGaN/AlGaN DAs | Pulse-2 | 20 | 15% |
| LED-2 | InGaN/AlGaN DAs | Pulse-3 | 5 |  |
| LED-3 | GaN | — | — |  |

1. **TEM image of DA-2**

**
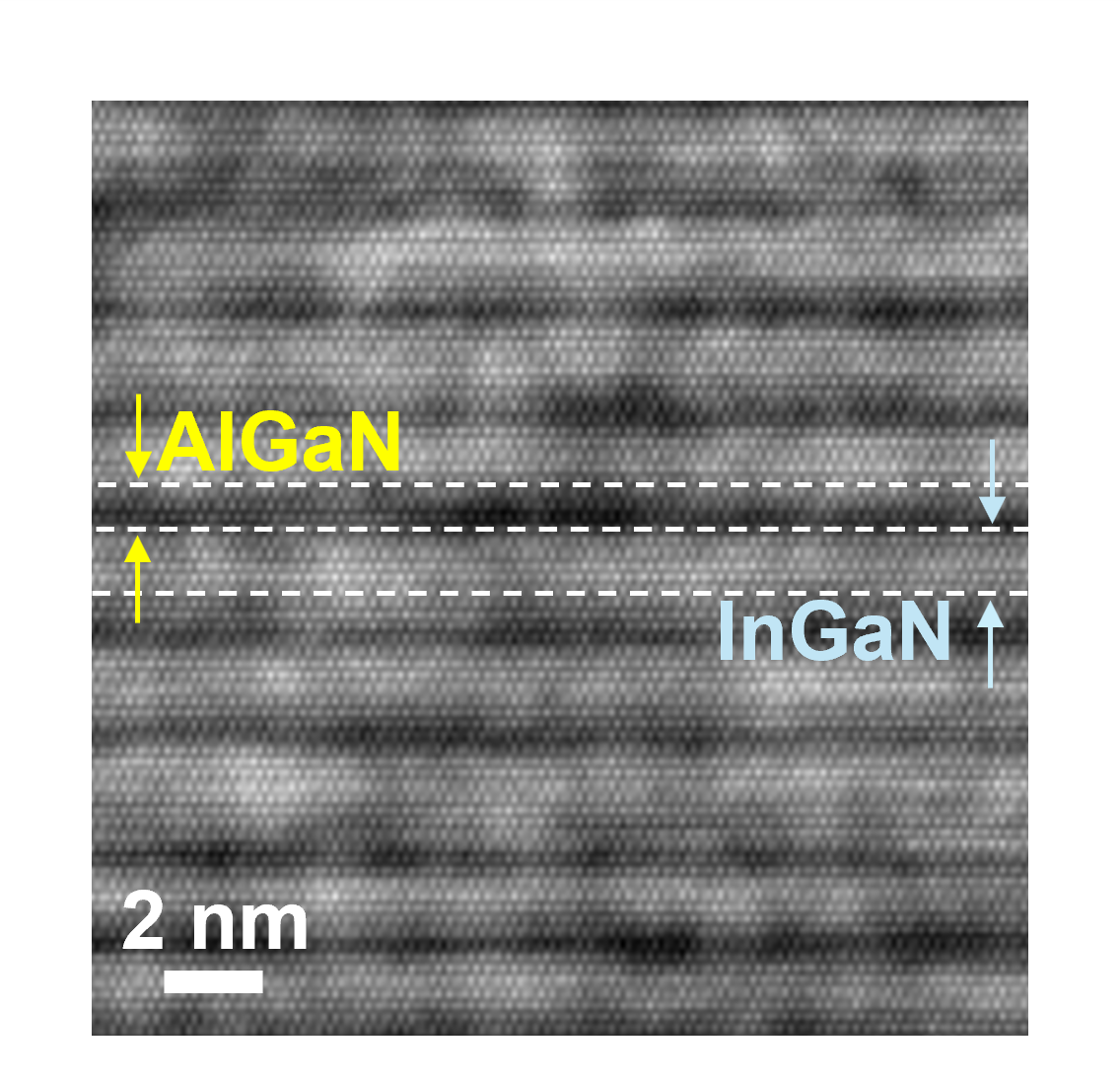
**

**Fig. S2.** TEM image of DA-2.

1. **Wafer wavelength mapping of LED-1, LED-2, and LED-3**

**
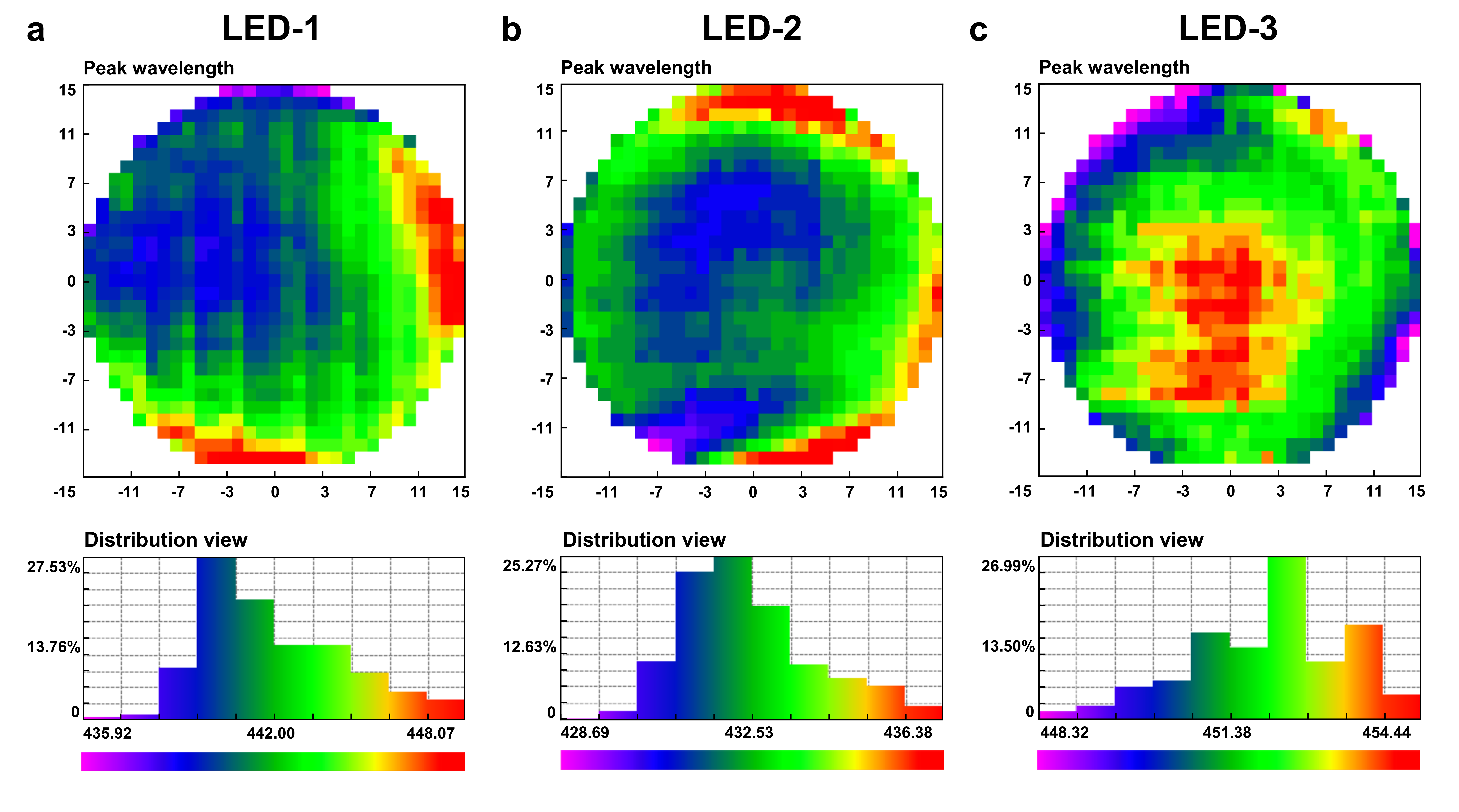
**

**Fig. S3.** Wafer wavelength mapping of **a** LED-1, **b** LED-2, and **c** LED-3.

1. **EL spectra, LOP, and EQE of LED-3**


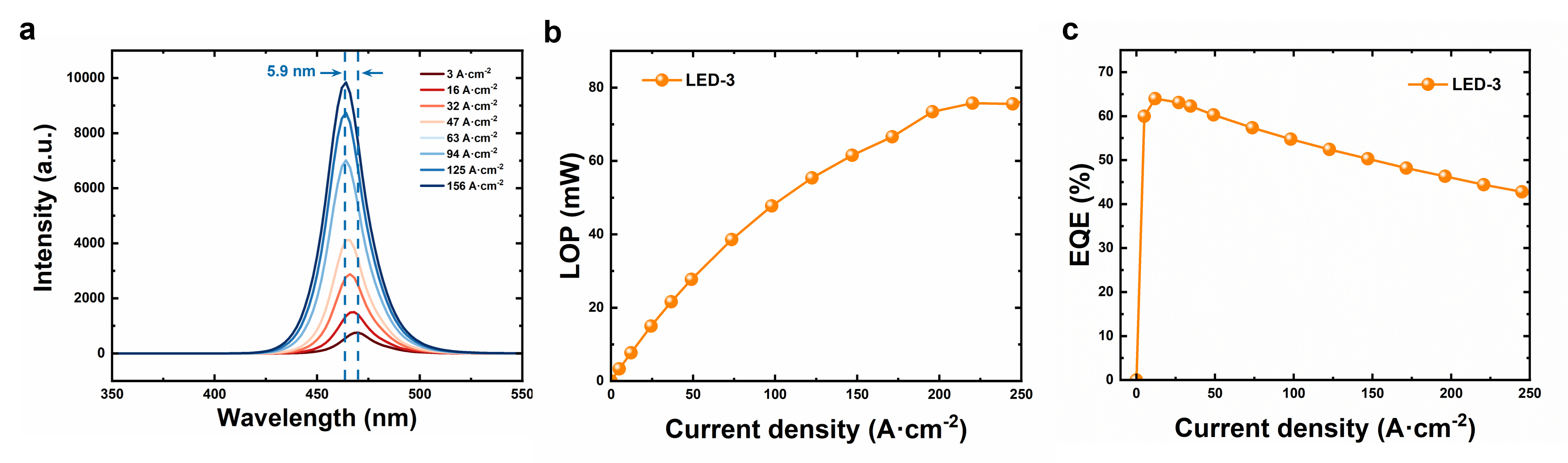


**Fig. S4. a** EL spectra of LED-3 at the current densities ranging from 3 A·cm^-2^ to 156 A·cm^-2^, **b** LOP and **c** EQE of LED-3 at the current densities ranging from 3 A·cm^-2^ to 250 A·cm^-2^.

1. **APSYS simulation of the lateral hole concentration distribution in QWs of LED-2 and LED-3**

**

**

**Fig. S5.** Simulated lateral hole concentration distribution in QWs of LED-2 and LED-3.

1. **Micro-PL images of LED-2 and LED-3**

Micro-photoluminescence (micro-PL) were performed on LED-2 and LED-3 to investigate the lateral carrier diffusion. The samples were excited by a laser emitting at 375 nm. The laser spot diameter of the 375 nm laser was approximately 5 μm. The emitted PL was collected by the microscope objective, as shown in Fig. S6. In addition, a long-pass filter was used to reject the laser wavelength. The micro-PL images were subsequently converted to grayscale for quantitative analysis of spot intensity distribution, as presented in Fig. S7. The color bar in the figure represents the normalized PL intensity (*I*_PL_).


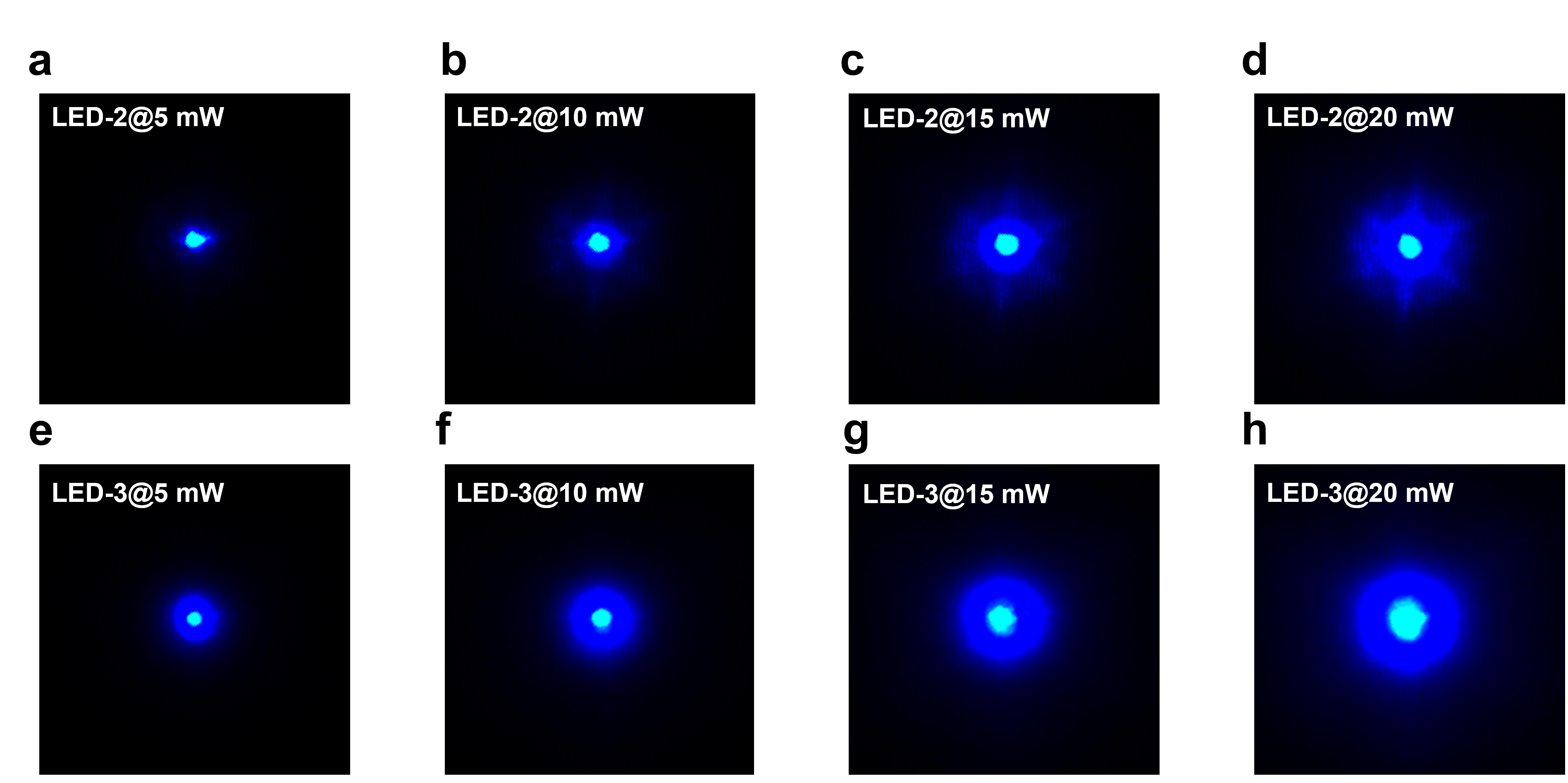


**Fig. S6.** Micro-PL images of LED-2 and LED-3 at the excitation power ranging from 5 mW to 20 mW.


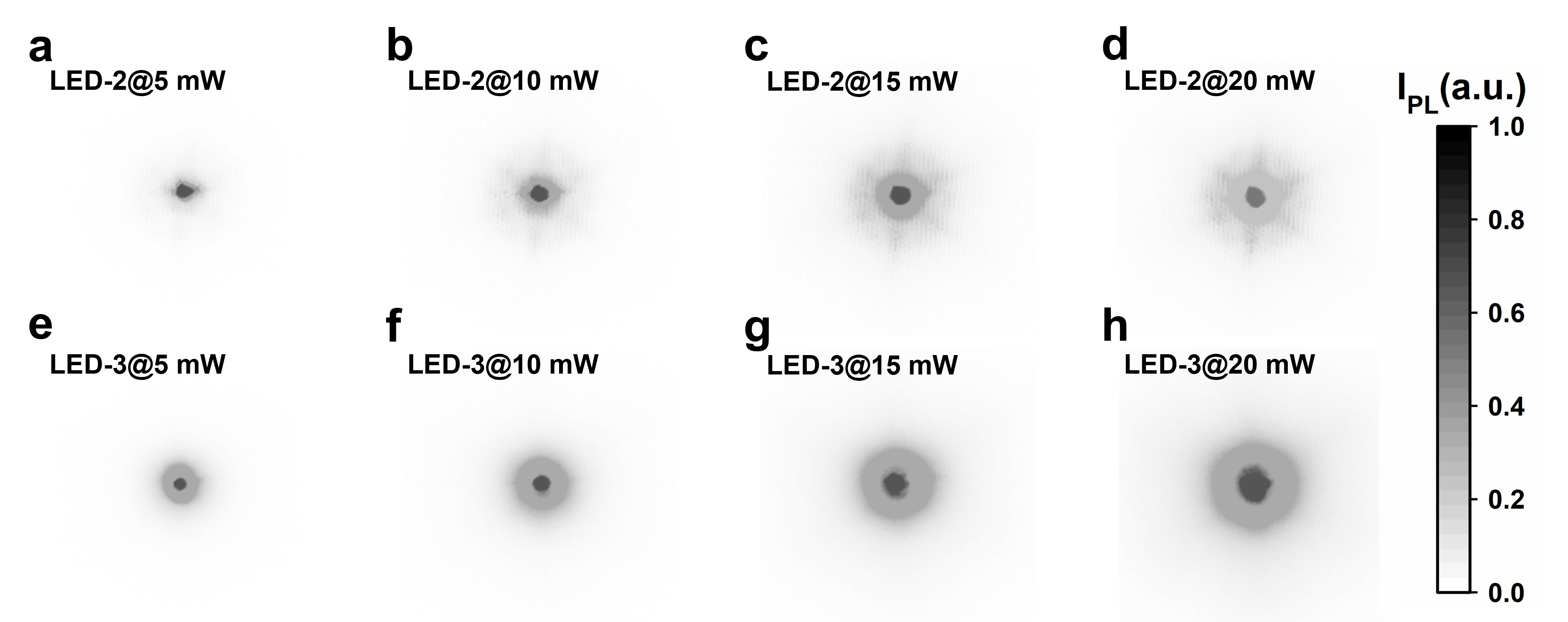


**Fig. S7.** Grayscaled micro-PL images of LED-2 and LED-3 at the excitation power ranging from 5 mW to 20 mW.

1. **Quantitative values of *J*_peak_ and *EQE*_peak_ of micro-LED2 and micro-LED3**

**Table S4.** Quantitative values of *J*_peak_ and *EQE*_peak_ of micro-LED2 and micro-LED3 for each size

| Size (μm^2^) | *l*/*S* (μm^-1^) | micro-LED2 | |  | micro-LED3 | |
| --- | --- | --- | --- | --- | --- | --- |
|  |  | *J*_peak_ (A·cm^-2^) | *EQE*_peak_ (%) |  | *J*_peak_ (A·cm^-2^) | *EQE*_peak_ (%) |
| 60×60 | 0.07 | 96 | 15.9 |  | 20 | 58.0 |
| 40×40 | 0.1 | 107 | 15.5 |  | 24 | 52.8 |
| 20×20 | 0.2 | 124 | 15.2 |  | 28 | 46.8 |
| 10×10 | 0.4 | 127 | 15.1 |  | 33 | 40.8 |

References for supplementary information

1. Schuster, M. et al. Determination of the chemical composition of distorted InGaN GaN heterostructures from x-ray diffraction data. Journal of Physics D-Applied Physics **32**, A56-A60 (1999).
